# Requirement of Interaction between Mast Cells and Skin Dendritic Cells to Establish Contact Hypersensitivity

**DOI:** 10.1371/journal.pone.0025538

**Published:** 2011-09-30

**Authors:** Atsushi Otsuka, Masato Kubo, Tetsuya Honda, Gyohei Egawa, Saeko Nakajima, Hideaki Tanizaki, Bongju Kim, Satoshi Matsuoka, Takeshi Watanabe, Susumu Nakae, Yoshiki Miyachi, Kenji Kabashima

**Affiliations:** 1 Department of Dermatology, Kyoto University Graduate School of Medicine, Kyoto, Japan; 2 Center for Innovation in Immunoregulative Technology and Therapeutics, Kyoto University Graduate School of Medicine, Kyoto, Japan; 3 Laboratory for Signal Network, Research Center for Allergy and Immunology, RIKEN Yokohama Institute, Tsurumi, Yokohama, Kanagawa, Japan; 4 Frontier Research Initiative, Institute of Medical Science, University of Tokyo, Minato, Tokyo, Japan; Tulane University, United States of America

## Abstract

The role of mast cells (MCs) in contact hypersensitivity (CHS) remains controversial. This is due in part to the use of the MC-deficient *Kit ^W/Wv^* mouse model, since *Kit ^W/Wv^* mice congenitally lack other types of cells as a result of a point mutation in c-kit. A recent study indicated that the intronic enhancer (IE) for *Il4* gene transcription is essential for MCs but not in other cell types. The aim of this study is to re-evaluate the roles of MCs in CHS using mice in which MCs can be conditionally and specifically depleted. Transgenic Mas-TRECK mice in which MCs are depleted conditionally were newly generated using cell-type specific gene regulation by IE. Using this mouse, CHS and FITC-induced cutaneous DC migration were analyzed. Chemotaxis assay and cytoplasmic Ca^2+^ imaging were performed by co-culture of bone marrow-derived MCs (BMMCs) and bone marrow-derived dendritic cells (BMDCs). In Mas-TRECK mice, CHS was attenuated when MCs were depleted during the sensitization phase. In addition, both maturation and migration of skin DCs were abrogated by MC depletion. Consistently, BMMCs enhanced maturation and chemotaxis of BMDC in ICAM-1 and TNF-α dependent manners Furthermore, stimulated BMDCs increased intracellular Ca^2+^ of MC upon direct interaction and up-regulated membrane-bound TNF-α on BMMCs. These results suggest that MCs enhance DC functions by interacting with DCs in the skin to establish the sensitization phase of CHS.

## Introduction

Contact hypersensitivity (CHS) has been widely used to study cutaneous immune responses, since it is a prototype of delayed-type hypersensitivity mediated by antigen -specific T cells [1], [2], [3], [4]. An essential step in the sensitization phase for CHS is the migration of hapten-bearing cutaneous dendritic cells (DCs), such as epidermal Langerhans cells (LCs) and dermal DCs, into skin-draining lymph nodes (LNs). After completing their maturation, mature DCs present antigen to naive T cells in the LNs, thus establishing the sensitization phase. In the subsequent challenge phase, re-exposure to the cognate hapten results in the recruitment of antigen-specific T cells and other non-antigen-specific leukocytes.

The functions of cutaneous DCs are modulated by keratinocyte-derived proinflammatory cytokines [1], [5]. The role of the different skin DC subsets in CHS (inducers, regulators, or functional redundancy) is a matter of active debate [6]. In addition, dermal DCs, including Langerin (CD207)^+^ dermal DCs, may also play an important role in CHS [7], [8].

Mast cells (MCs) are a candidate DC modulator since they express and release a wide variety of intermediaries, such as histamine, tumor necrosis factor (TNF)-α and lipid mediators. It has been reported that activated human cord blood-derived MCs induce DC maturation *in vitro* [9], that IgE-stimulated MC-derived histamine induces murine LC migration *in vivo* [10], and that MC-derived TNF-α promotes cutaneous murine DC migration *in vivo* in an IgE-independent manner [11]. On the other hand, prostaglandin (PG) D_2_ produced by MCs in response to allergens [12], inhibits LC migration [13]. Therefore, MCs might have bi-directional effects on DC activity in a context-dependent manner and the question of the mechanisms by which DCs are modulated by MCs is an important issue to pursue.

While MCs have been assumed to play an important role in CHS, their role is controversial. Previous studies have demonstrated that MC-deficient *Kit ^W/Wv^* mice show attenuated CHS responses, meanwhile, other studies have shown that CHS was not impaired in *Kit^W/Wv^* mice [14]. Although some studies indicated that the discrepancy in *W/Wv* mice might be due to the difference in hapten dose, the detailed mechanism is still unclear. *Kit ^W/Wv^* mice and *Kit^W-sh/KitW-sh^* mice have an inversion mutation in the Kit gene [15], and therefore, these mice also lack melanocytes and hematopoietic stem cells, which are known to modulate immune responses [16], [17]. In addition, since MCs are congenitally absent, it is possible that compensatory mechanisms may exist that modulates immune system functions. Therefore, it is important to re-evaluate the roles of MCs using mice in which MCs can be conditionally and specifically depleted.

Recently, we have demonstrated that MCs and basophils use specific enhancer elements, intronic enhancer (IE) and a 3′ 4kb fragment that contains 3′UTR and HS4 elements, to regulate *Il4* gene expression, respectively [18]. Taking advantage of this system, we have generated mice that contain human diphtheria toxin receptor (DTR) under the control of IE. Therefore, mast cell-specific enhancer-mediated Toxin Receptor-mediated Conditional cell Knock out (TRECK) systems were designated as Mas-TRECK transgenic (Tg) mice. In these mice, both MCs and basophils are conditionally depleted by diphtheria toxin (DT) treatment. Since basophils recover much faster than MCs (Fig S1A, B), there exist a period of specific MC depletion. Taking advantage of the system, we have herein demonstrated that activated DCs induce MC activation, which triggers the migration and maturation of DCs via cell-cell contact. This DC-MC interaction plays an essential role in the sensitization phase of CHS.

## Results

### Suppression of CHS responses in Mas-TRECK Tg mice

Mice expressing the human DTR under the control of IE element (for Mas-TRECK) and 3′UTR element (for basophil-specific enhancer-mediated TRECK systems; Bas-TRECK) in the *Il4* gene locus were generated by a transgenic strategy (Sawaguchi et al. Manuscript in submission). We initially demonstrated that skin MCs were completely depleted in Mas-TRECK Tg mice 5 and 12 days after an intraperitoneal injection of diphtheria toxin (DT) (See **Fig. S1A** in the Online Repository). Although DX5+ FcεRIα+ basophils in the blood were eliminated 5 days after DT treatment in Mas-TRECK Tg mice, basophil numbers recovered in 12 days (**Fig. S1B** in the Online Repository).

**Figure 1:**
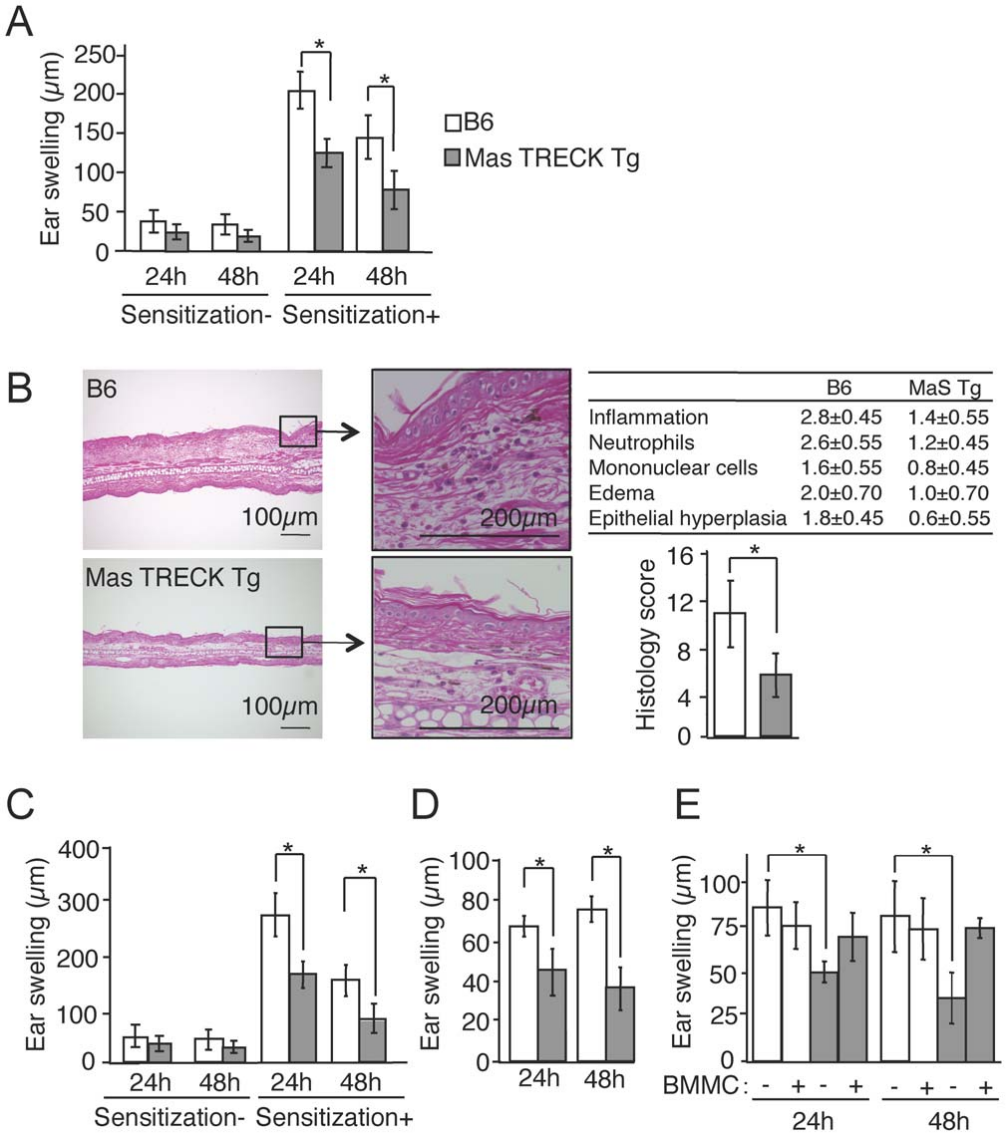
MCs are essential during the sensitization phase in CHS. (A) Twelve days after DT treatment, WT and MaS TRECK Tg mice (*n* = 12 per group) were sensitized with or without DNFB and the ear swelling was measured 24 and 48 h after challenge with DNFB. (B) HE staining of the ears of sensitized DT-treated WT and MaS TRECK Tg mice 24 h after challenge with DNFB. Scale bar, 100 µm (left panels) and 200 µm (middle panels). Samples were scored for the severity and character of the inflammatory response using a subjective grading scale. The total histology score was calculated as the sum of scores (right panels). (C) Oxazolone-induced CHS in WT (white columns) and Mas-TRECK Tg (grey columns) mice. DT-treated WT and MaS TRECK Tg mice (*n* = 13 per group) were sensitized with oxazolone and ear swelling measured 24 and 48 hours after challenge with oxazolone. (D) CHS induced by adoptive transfer of LN cells sensitized with DNFB of WT (white columns) and Mas-TRECK Tg (grey columns) (*n* = 6 per group). (E) Draining LNs from oxazolone-sensitized WT (white columns) and Mas-TRECK Tg (grey columns) reconstituted with BMMCs (*n* = 5 per group) were adoptively transferred to induce CHS. All data are presented as the mean ± SD and are representative of three experiments. ^*^, *P*<0.05 versus corresponding mice.

To investigate the role of MCs in cutaneous acquired immune responses, we used DNFB-induced CHS as a model. CHS responses were similar between wild type (WT) and Mas-TRECK Tg mice in the absence of DT treatment (205 µm±10.5 vs 212 µm±12.3; average ± SD). In addition, DT treatment itself did not affect the degree of CHS responses in WT mice. On the other hand, when both WT and Mas-TRECK Tg mice were treated with DT and assayed 12 days later, the CHS response in Mas-TRECK Tg mice was much less than that in WT mice (**Fig. 1A**). The ear swelling of WT and Mas TRECK Tg mice was 48.2 (±5.2, SD) µm and 51.3 (±4.8, SD) µ after 72 h, and 15 (±7.29, SD) µm and 40.1 (±6.68, SD) µm after 96 h respectively. Histology of the ears 48 h after the challenge showed considerable lymphocyte infiltration and edema in the dermis of sensitized WT mice; these changes were less apparent in Mas-TRECK Tg mice (**Fig. 1B, *left panel***) and the histological scores [19] in Mas-TRECK Tg mice were lower than those in WT mice (**Fig. 1B, *right panel***). On the other hand, the CHS response was not impaired in ***Kit^W/Wv^*** mice (See **Fig. S2A** in the Online Repository).

In addition, the CHS response in Bas-TRECK Tg mice, which lack only basophils upon treatment with DT, was similar to that of WT mice (See **Fig. S2B** in the Online Repository). The attenuated CHS response in Mas-TRECK Tg mice was confirmed using an additional hapten, oxazolone (**Fig. 1C**) and also at higher hapten doses (See **Fig. S2C** in the Online Repository).

To clarify the action phase of MCs in CHS, we used an adoptive transfer-induced CHS model. Recipients of LN cells from sensitized WT mice showed an enhanced CHS response, whereas the recipients of LN cells from sensitized Mas-TRECK Tg mice showed a markedly inhibited response (**Fig. 1D**). In addition, the recipients of CD90.2+ T cells from sensitized Mas-TRECK Tg mice showed inhibited responses compared to recipients of CD90.2+ T cells from sensitized WT mice (See **Fig. S2D** in the Online Repository). These data indicate that MCs play important roles in establishing CHS during the sensitization phase.

We further evaluated whether the attenuated CHS in Mas-TRECK Tg mice reflected the lack of MCs. WT or Mas-TRECK Tg mice were engrafted in the skin with or without BMMCs 5×10^6^ cells in 100 µl/dorsal skin 1 hour before oxazolone sensitization. The numbers of toluidine blue positive mast cells (per field) in the dermis are 40.2 (±5.3, SD) in Mas TRECK Tg mice and 35.3 (±7.2, SD) in B6 wild type mice after one hour injection of BMMC (n = 3) (See **Fig. S2E** in the Online Repository). On the other hand, the number of MCs in the uninjected sites was 10.5 (±3.2, SD) in B6 wild type mice. Five days after sensitization, the skin-draining LN cells of sensitized mice were adoptively transferred intravenously into naive WT recipients and challenged with oxazolone on the ears. The CHS response of recipients of LN cells from sensitized WT mice was not changed by the pre-engraftment of BMMCs into the skin (**Fig. 1E**). On the other hand, the attenuated CHS response of recipients of Mas-TRECK Tg LN cells was fully restored by the pre-engraftment of BMMCs into the skin.

Next we counted the cells infiltrating the skin of WT and Mas-TRECK Tg mice 12 h after challenge with DNFB. The numbers of CD45+ CD3+ CD4+ T cells, CD45+ CD3+ CD8+ T cells, and CD45+ Gr-1high neutrophils after both sensitization and challenge, and that of neutrophils after only challenge were increased in WT mice. But such increment was attenuated in Mas-TRECK Tg mice (See **Fig. S3A** in the Online Repository). Consistent with this result, the mRNA levels of IFN-γ, IL-17 and IL-1β in the skin 12 h after challenge were significantly decreased in Mas-TRECK Tg mice compared to WT mice (See **Fig. S3B** in the Online Repository).

We further analyzed the composition of LN cells after sensitization. Five days after sensitization, the skin-draining LN cells of WT and Mas-TRECK Tg mice were collected. The numbers of CD44+ CD62L+ central memory T cells and CD44+ CD62L- effector memory T cells among CD4+ and CD8+ T cell subsets were less in Mas-TRECK Tg mice than in WT mice (See **Fig. S4A** in the Online Repository). In contrast, the numbers within each subset in the LN without sensitization were comparable between WT and Mas-TRECK Tg mice (See **Fig. S4A** in the Online Repository).

To evaluate of T cell differentiation after sensitization, the skin-draining LN cells from control or DNFB-sensitized WT and Mas-TRECK Tg mice were challenged in the presence or absence of DNBS *in vitro*. The incorporation of ^3^H-thymidine and the levels of IFN-γ and IL-17 in the culture supernatant in the presence of DNBS were markedly decreased in LN cells from Mas-TRECK Tg mice as compared with those from WT mice (See **Fig. S4B-D** in the Online Repository). The levels of IL-4 in the culture supernatants were below the limit of detection of ELISA (<0.3 pg/mL).

### Attenuated DC migration and maturation in the skin-draining LNs of Mas-TRECK Tg mice

An essential step in the sensitization phase for CHS is the migration of hapten-bearing cutaneous dendritic cells (DCs), such as epidermal Langerhans cells (LCs) and dermal DCs, into skin-draining lymph nodes (LNs). Accordingly, to dissect the site of action of MCs in the sensitization phase, we initially focused on cutaneous DCs that have an opportunity to interact with MCs present in the dermis.

**Figure 2:**
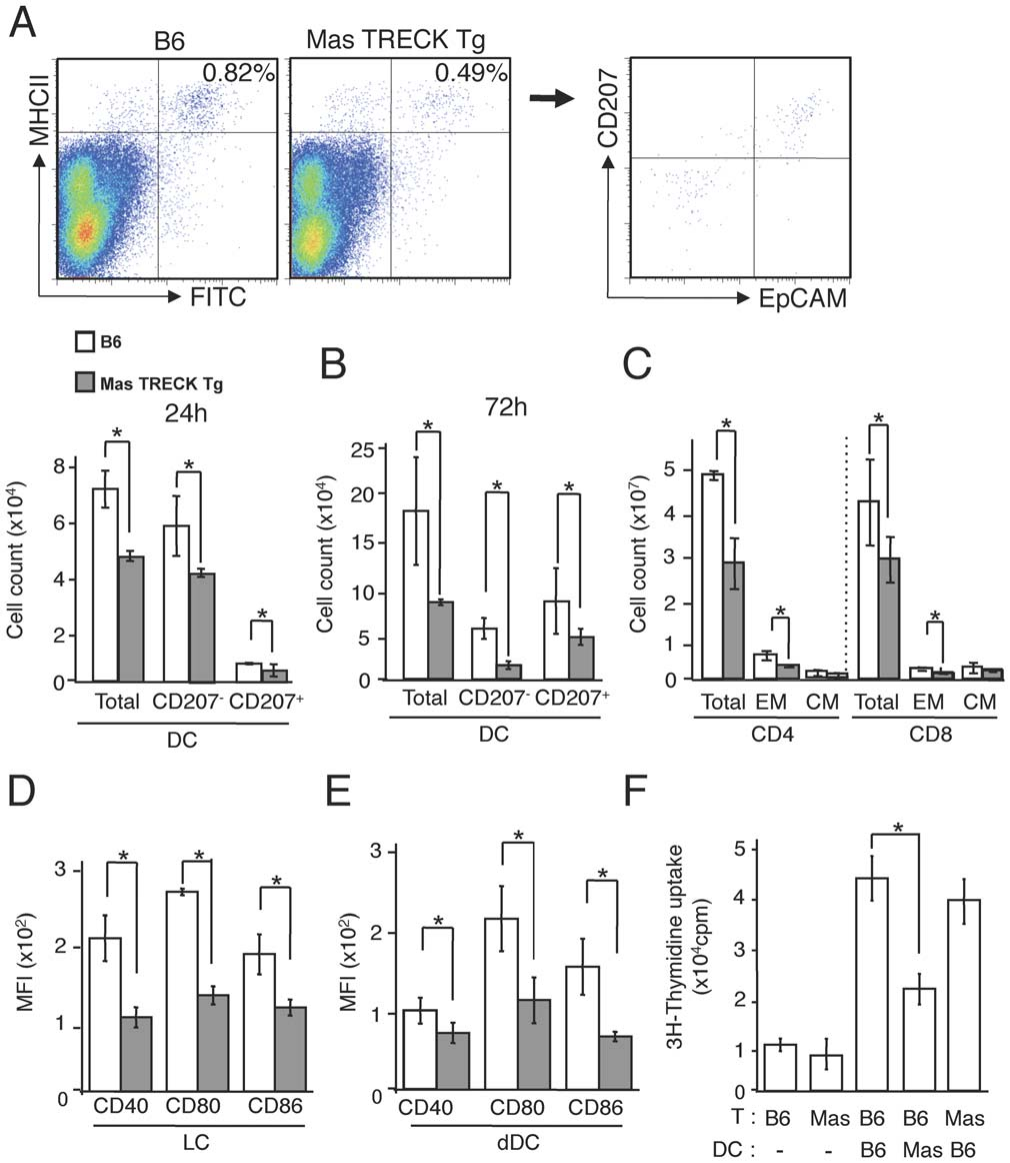
Impaired cutaneous DC migration and maturation in Mas-TRECK Tg mice. (A, B) The numbers of FITC^+^ CD11c^+^ MHC class II^+^ CD207^+^ DCs and FITC^+^ CD11c^+^ MHC class II^+^ CD207^−^ DCs in the draining LNs of DT-treated MaS TRECK Tg and WT mice 24 h and 72 h after application of FITC. (**C**) The numbers of total, central memory (CM), and effector memory (EM) CD4^+^ and CD8^+^ T cells in the draining LN 72 h after FITC application are shown. (D, E) The expression levels of CD40, CD80, and CD86 on both LCs and dDCs of DT-treated MaS TRECK Tg and WT mice. (F) *In vitro* assay of T-cell proliferation induced by DCs sorted from the draining LN of sensitized mice of WT or Mas-TRECK Tg (Mas) mice. Oxazolone-sensitized CD90.2^+^ T cells were purified from the draining LNs of WT or Mas-TRECK Tg (Mas) mice 5 d after oxazolone application. T cells (5×10^5^ cells) were incubated for 72 h, pulsed with 0.5 µCi [^3^H]thymidine for the last 24 h ,with or without CD11c^+^ DCs (1×10^5^ cells) prepared from the draining LNs of DT-treated WT and MaS TRECK Tg mice one day after oxazolone application. All data are presented as the mean ± SD and are representative of three experiments. ^*^, *P*<0.05 versus a corresponding group.

Using a FITC-induced cutaneous DC migration model, we found that the numbers of both FITC^+^ CD11c^+^ MHC class II^+^ CD207^+^ DCs and FITC^+^ CD11c^+^ MHC class II^+^ CD207^−^ DCs in the draining LNs 24 h and 72 h after FITC application were significantly attenuated in Mas-TRECK Tg mice compared to WT mice (**Fig. 2A, B**). In addition, the numbers of total CD4^+^ and CD8^+^ T cells, and CD44^+^ CD62L^+^ central memory and CD44^+^ CD62L^−^ effector memory T cells in the draining LNs of Mas-TRECK Tg mice were less than those of WT mice (**Fig. 2C**). We next analyzed the expression levels of costimulatory molecules by skin organ culture. We incubated the skin and analyzed the expression levels on crawl-out DCs in the culture medium. The expression levels of CD40, CD80, and CD86 both on CD11c^+^ MHC class II^+^ EpCAM^+^ LCs and CD11c^+^ MHC class II^+^ EpCAM^−^ dermal DCs in Mas-TRECK Tg mice were lower than those of WT mice (**Fig. 2D, E** **,** and see **Fig. S5A**, upper panel, in the Online Repository). On the other hand, the expression levels of costimulatory molecules on LCs and dermal DCs from the untreated WT controls and Mas TRECK Tg mice were comparable (See **Fig. S5A**, lower panel, in the Online Repository). In addition, the expression levels on LCs and dermal DCs from WT and Mas-TRECK mice were comparable under steady state conditions (See **Fig. S5B** in the Online Repository). Consistently, LCs and dermal DCs from *Kit+/+* mice were similar to those from *Kit ^W/Wv^* mice (See **Fig. S5B** in the Online Repository).

We further evaluated the effect of MCs on the antigen presenting capacity of DCs. We sorted 5×10^5^ T cells by auto MACS from the draining LNs of CD90.2^+^ WT mice five days after 25 µl of 2% oxazolone application. These CD90.2^+^ T cells were incubated for 72 h with or without CD11c^+^ DCs (1×10^5^ cells) prepared from the draining LNs of WT or Mas-TRECK Tg mice one day after oxazolone application. T cell proliferation was enhanced by the addition of antigen-acquired DCs sorted from the draining LN of sensitized mice, and the extent of augmentation by DCs from WT mice was much higher than that of DCs from Mas-TRECK Tg mice (**Fig. 2F**).

### Enhancement of BMDC maturation and chemotaxis by BMMC requires cell-cell contact in vitro

**Figure 3:**
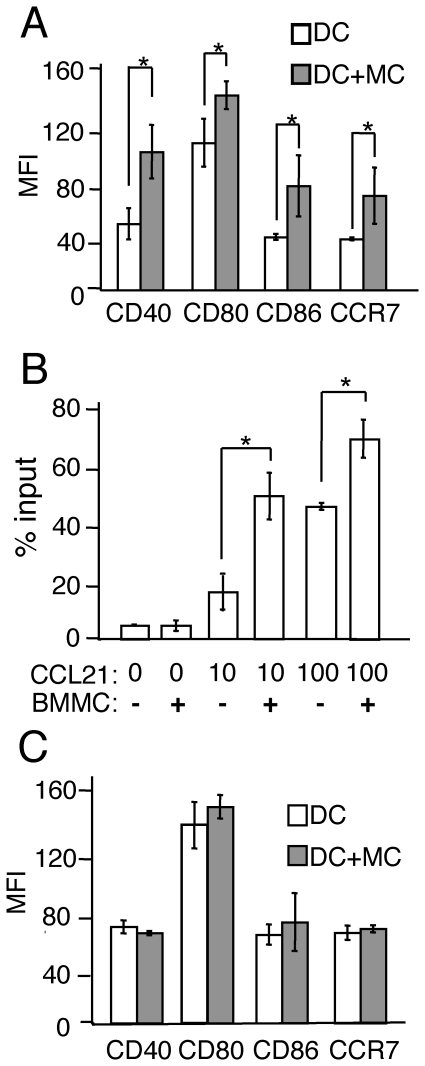
BMMCs promote the maturation and chemotactic activity of BMDCs via direct cell interaction. (A) The expression levels of CD40, CD80, CD86 and CCR7 on BMDCs cultured with or without BMMCs for 24 h. (B) Mobility of BMMCs to CCL21. BMMCs with or without BMDCs were applied to the upper chamber of a transwell without coating for 3 h. The numbers of MHC class II^+^ cells in the lower chamber, identified as migrating DCs, were counted by means of flow cytometry. (C) The expression levels of CD40, CD80, CD86 and CCR7 on BMDCs cultured with or without BMMCs separately by using transwell culture plates for 24 h. All data are presented as the mean ± SD and are representative of three experiments. ^*^, *P*<0.05 versus a corresponding group.

Impairment of DC functions as a result of MC deficiency suggests that MCs stimulate cutaneous DCs. To address this hypothesis, we prepared BMDCs [20] and incubated them with or without BMMCs. Co-cultivation of BMDCs with BMMCs for 24 h significantly increased the expression levels of CD40, CD80, CD86 and CCR7 on BMDCs (**Fig. 3A**). In addition the chemotaxis of BMDCs to CCL21 was significantly enhanced, when BMMCs were added to the upper chamber with BMDCs (**Fig. 3B**).

Furthermore addition of BMMCs to the upper chamber of transwells did not induce further up-regulation of CD40, CD80, CD86 and CCR7 levels on BMDCs incubated in the lower chamber (**Fig. 3C**), which suggests that BMMCs require direct cell-cell interaction to stimulate BMDCs.

### Stimulation of BMMCs by activated BMDCs

**Figure 4:**
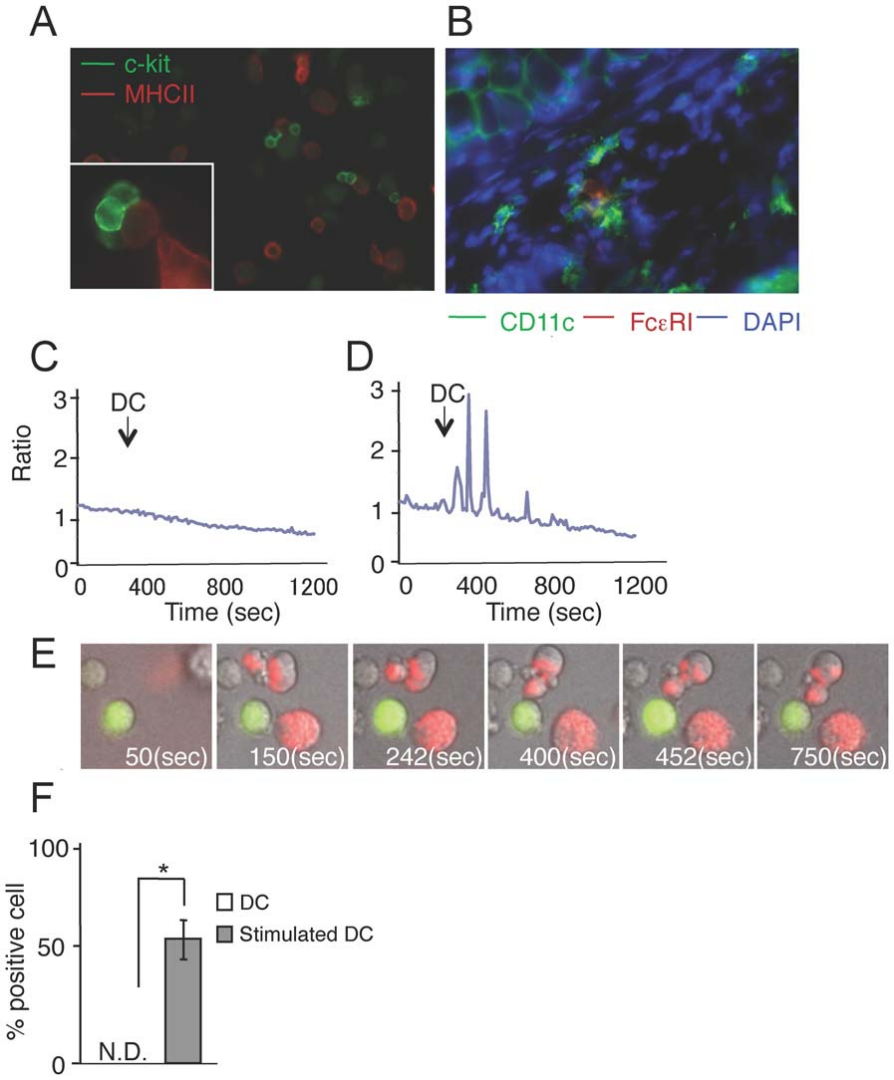
Stimulated BMDCs enhance Ca^2+^ influx in BMMCs and BMMCs interact with DCs in vitro and in vivo. (A) On poly-L-lysine coated glass coverslips, c-kit^+^ BMMCs (green) contacted MHC class II^+^ BMDCs (red). (B) Conjugate formation of CD11c^+^ DCs (green) with FcεRIα^+^ MCs (red) in the sensitized skin. (C–E) BMMCs (green) were co-cultured with non-stimulated BMDCs (C) or BMDCs stimulated with 100 ng/ml of LPS and 50 ng/ml of CCL21 for 1 h (red; D, E), and the intracellular Ca^2+^ was monitored by Fluo-8 for 1200 sec. Photos were taken at the indicated time points from Supplemental Movie 2 (E). (F) The percentage of BMMCs demonstrating high Ca^2+^ concentration among BMMCs. Data are presented as the mean ± SD and are representative of three experiments. ^*^, *P*<0.05 versus a corresponding group.

We then examined *in vitro* whether DCs directly contacted MCs. We incubated BMMCs and BMDCs, and found that c-kit^+^ BMMCs contacted MHC class II^+^ BMDCs (**Fig. 4A**). A number of FcεRIα^+^ CD11c^−^ MCs co-localized with CD11c^+^ DCs in ear dermis 24 h after sensitization with DNFB (**Fig. 4B**). Consistent with this, the number of MCs co-located with DCs after sensitization was higher than that in the steady state (in other words, in non-inflammatory conditions) (4±0.81 vs 0.3±0.58; average ± SD).

At present, several stimuli in addition to IgE are known to trigger calcium influx and activate MCs [21]. Therefore, we studied the effect of BMDCs on BMMCs using Ca^2+^ imaging. We incubated tetramethylrhodamine ethyl ester (TMRE)-labeled BMDCs and Fluo-8-labeled BMMCs together. When an intracellular Ca^2+^ concentration of Fluo-8 (green) -labeled BMMCs is upregulated, fluorescence intensity of green becomes increased. Unstimulated CD11c^+^ MHC class II^int+^ BMDCs did not increase BMMC intracellular Ca^2+^ concentrations (**Fig. 4C** **,** See **Video S1** in the Online Repository). On the other hand, stimulated CD11c^+^ MHC class II^high+^ BMDCs induced prominent Ca^2+^ increase in BMMCs (**Fig. 4D** and See **Video S2** in the Online Repository). This rapid rise occurred five to ten times in 1000 seconds, and each spike lasted about 10–20 sec (**Fig. 4E**). Stimulated BMDCs significantly upregulated the ratio of BMMCs with increased Ca^2+^ concentration compared to non-stimulated BMDCs (**Fig. 4F**). These results suggest that stimulated DCs activate MCs via direct cell-cell contact.

### Stimulation of DCs by MCs is dependent on ICAM-1-LFA-1 interaction and on MC membrane-bound TNF-α

**Figure 5:**
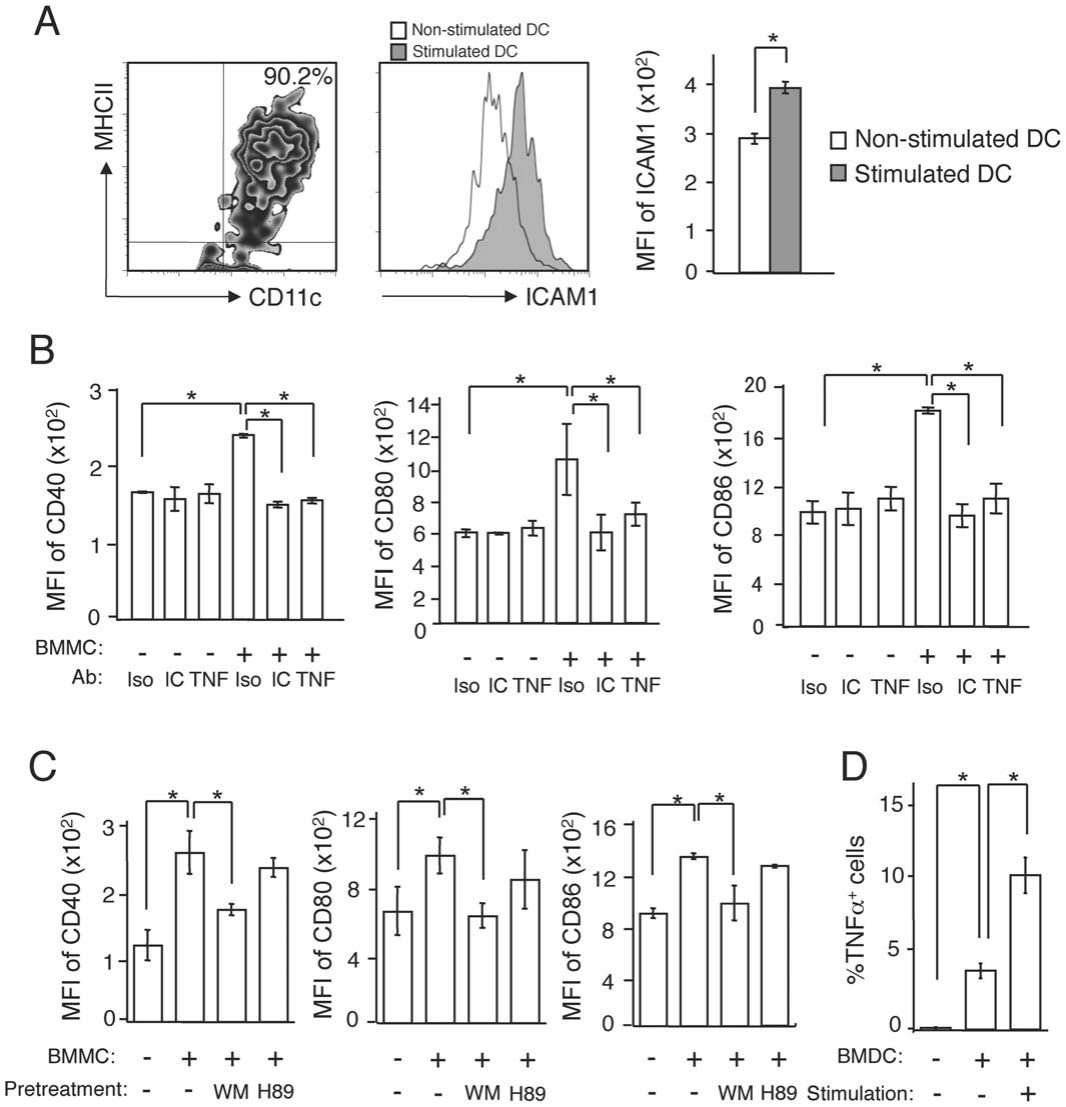
DC stimulation depends on ICAM-1-LFA-1 signals and membrane-bound TNF-α on MCs. (A) The expression of ICAM-1 on stimulated or non-stimulated BMDCs. (B, C) The expression levels of CD40, CD80, and CD86 on BMDCs co-cultured with BMMCs with isotype control Ab (Iso), anti-ICAM-1 Ab (IC), or anti-TNF-α Ab (TNF) (B), or co-cultured with BMMCs that were pretreated with or without wortmannin (WM) or H89 (C). (D) BMMCs expressing membrane-bound TNF-α in the presence of absence of BMDCs with or without stimulation with 100 ng/ml of LPS and 50 ng/ml of CCL21 for 1 h. All data are presented as the mean ± SD and are representative of three experiments. ^*^, *P*<0.05.

We then sought to identify how MCs promote DC maturation. It has been reported that ICAM-1 on the surface of MCs directly interacts with leukocyte function-associated antigen 1 (LFA-1) on T cells, and that stimuli such as CD40L, LPS, and TNF-α, upregulate the expression of ICAM-1 on DCs [22]. In fact, the expression of ICAM-1 on BMDCs was up-regulated upon stimulation of BMDCs by LPS and CCL21 (**Fig. 5A**). Therefore, we hypothesized that MCs and DCs interact in an ICAM-1- and LFA-1-dependent manner. Addition of neutralizing anti-ICAM-1 antibody to a culture of BMDCs completely inhibited upregulation of CD40, CD80 and CD86 expression on BMDCs upon addition of BMMC (**Fig. 5B** **, S6A**).

We then analyzed intracellular signaling using the protein kinase A inhibitor, H89, and the phosphoinositide 3 kinase inhibitor, wortmannin. Although H89 did not inhibit the BMMC-induced upregulation of co-stimulatory molecules on BMDCs, wortmannin inhibited DC maturation (**Fig. 5C** **, S6B**). These results suggest that binding of ICAM-1 to LFA-1 activates DCs through a PI3-kinase pathway.

Lastly we tried to identify how DCs induce MC activation. TNF-α is first produced as a 26 kDa transmembrane molecule (membrane-bound TNF-α), which is cleaved by the metalloproteinase-disintegrin TNF-α converting enzyme TACE [23] to generate a soluble 17 kDa TNF-α. Studies have shown that membrane-bound TNF-α is also biologically active [24]. We first observed that anti-TNF-α antibody completely blocked DC maturation induced by BMMCs (**Fig. 5B**). Consistently, BMMC from TNF-α KO mice did not promote BMDC maturation (**Fig. S6C**). But the soluble form of TNF-α in the supernatant of co-cultures of BMMCs and TNF-α KO-derived BMDCs was below the detection limit irrespective of BMDC stimulation (<8 pg/ml, each). On the other hand, the level of membrane-bound TNF-α on BMMCs was increased by the addition of BMDCs and even further enhanced when stimulated BMDCs were added (**Fig. 5D**). These results suggest that MCs express membrane-bound TNF-α upon direct interaction with activated DCs through ICAM-1 on DCs and that membrane-bound TNF-α induces expression of co-stimulatory molecules on DCs.

## Discussion

In this study, we used Mas-TRECK Tg mice in which MCs can be eliminated specifically and conditionally, and demonstrated that CHS was significantly attenuated in accord with impaired memory T cell induction in skin-draining LNs after sensitization. MC depletion also impaired hapten-induced cutaneous DC migration concordant with levels of co-stimulatory molecule expression. In addition, BMMCs promoted BMDC maturation and chemotactic activity by direct interaction via ICAM-1 and membrane-bound TNF-α. In fact, a certain number of DCs were found colocalized with MCs in the DNFB-sensitized dermis. These findings suggest that MCs may promote migration and maturation of dermal DCs and epidermal LCs to establish the sensitization phase of CHS.

It was previously reported that FITC-induced cutaneous DC migration was attenuated in *Kit^W-sh/KitW-sh^* mice at 24 h, but not at 48 or 72 h after FITC application, and that the sensitization phase of CHS was not attenuated in *Kit^W-sh/KitW-sh^* mice [11]. The above findings were inconsistent with our findings in the way that cutaneous DC migration was attenuated even at 72 h after FITC application and that sensitization phase was impaired in Mas-TRECK Tg mice. On the other hand, it has also been shown that MCs are capable of influence both the sensitization phase and the elicitation phase in other models of CHS [25]. Therefore, it still remains unknown how the discrepancy occurred. The difference between our model and *Kit ^W/Wv^* and *Kit^W-sh/KitW-sh^* mice is the existence of melanocytes and hematopoietic stem cells. Recently, melanocytes were shown to express toll like receptors, to modulate immune responses and to produce IL-1a and IL-1b [16], [17]. In addition, because of congenital absence of MCs in *Kit ^W/Wv^* and *Kit^W-sh/KitW-sh^* mice, compensatory mechanism may exist, such as repopulation of basophils. In fact, the numbers of basophils have been counted in these mice; the number of basophils in in *Kit^ W/Wv^* mice are lower than that in WT mice, and that in *Kit^W-sh/KitW-sh^* mice are higher than that in WT mice [26]. These results indicate that the compensatory mechanisms may affect the result of CHS responses and that *Kit ^W/Wv^* and *Kit^W-sh/KitW-sh^* mice may not necessarily be representative to evaluate the roles of pure MCs. In this study, we have demonstrated that the attenuated CHS response in Mas TRECK Tg mice was fully restored by the pre-engraftment of BMMCs into the skin just before sensitization with hapten. It is intriguing to evaluate this recovery by means of reconstitution with native skin MCs, since freshly generated BMMC and native skin MCs can differ in aspects of phenotype and function. We can't perform long term engraftment of BMMC in this model because we have found that repeated DT treatment (more than 5 days of daily injection) leads to weak viability of the Mas TRECK Tg mice.

We showed that co-culture of DCs with MCs did not promote soluble TNF-α secretion from MCs but up-regulated membrane-bound TNF-α on MCs. Since TNF-α expressed on MCs in this context is membrane-bound, MCs are required to co-localize with DCs *in vivo* to elicit its effect. Since neutralizing anti-TNF-α Ab abrogated the BMMC-induced DC maturation, membrane-bound TNF-α on MCs might be the major modulator of DC maturation. Herein we have focused on the roles of MCs in the sensitization phase, but we also noted that MC depletion in the elicitation phase attenuated the CHS response. Since DCs are thought to present antigen to memory T cells to initiate the challenge phase of CHS, the interaction of MCs and DCs might be essential for its establishment, which is a question that will be pursued in a future study.

The direct interaction between DCs and MCs was essential not only for DC stimulation, but also for MC activation. Previous reports have demonstrated that MCs contact extracellular matrix components to provide a co-stimulatory signal for histamine and cytokine production via integrins [27]. MCs are known to degranulate upon binding of ICAM-1 on MCs and LFA-1 on activated T cells [28]. In agreement with this, our study demonstrates that the interaction of MCs with DCs is dependent on cell-cell contact via ICAM-1 and LFA-1. In addition, an influx of calcium in MCs is induced by activated DCs that express high levels of ICAM-1, but not by immature DCs. Therefore, in sensitized skin, activated DCs bind to MCs and promote activation and up-regulating membrane-bound TNF-α on MCs. Activated MCs further promote additional activation of hapten-bearing cutaneous DCs causing them to migrate into skin-draining LNs. These findings indicate that MCs play an essential role in establishing the sensitization phase of CHS by promoting cutaneous DC functions.

## Materials and Methods

### Mice

Mice expressing the human DTR under the control of IE element (for Mas-TRECK) and 3′UTR element (for Bas-TRECK) in the *Il4* locus were generated by a transgenic strategy. The basic pIL-4 construct was made by insertion of the 5′enhancer (5′E) (−863 to −5448; start codon is defined as sequence number 0) [29] and the IL-4 promoter from position −64 to −827. Human DTR fragment was isolated from human DTR/pMS7 vector that was provided by Dr. M. Tanaka (RCAI, RIKEN, Yokohama, Japan) and inserted into the basic pIL-4 construct. IE (+311 to +3534) and 3′UTR (+6231 to +10678) fragments were isolated from a mouse YAC clone (catalog no. 95022; Research Genetics, Huntsville, AL) and inserted into the basic pIL-4 and human DTR construct respectively. Each transgenic (Tg) line was generated on a C57BL/6 background. C57BL/6 (B6, WT) mice were purchased from Japan SLC (Shizuoka, Japan). TNF-α KO mice on the C57BL/6 background were generated [30]. WBB6F1-*Kit*+*/*+ and –*Kit ^W/Wv^* mice were obtained from The Jackson Laboratory. For DT treatment, mice were injected intraperitoneally with 250 ng of DT in 250 µl of PBS per mouse for five consecutive days. Eight to ten week-old female mice were used for all the experiments and bred in specific pathogen-free facilities at Kyoto University. All experimental procedures were approved by the institutional animal care and use committee of Kyoto University Graduate School of Medicine (MedKyo11100).

### Reagents, antibodies and intracellular staining

We purchased dinitrofluorobenzene (DNFB) from Nacalai Tesque (Kyoto, Japan), 2,4- dinitrobenzene sulfonic acid (DNBS) from Alfa Aesar (Ward Hill, MA). FITC-, PE-, PE-Cy5-, PE-Cy7-, APC-, APC-7-, and Pacific Blue-conjugated 145-2C11 (anti-CD3), GK1.5 (anti-CD4), 53-6.7 (anti-CD8), N418 (anti-CD11c), 1C10 (anti-CD40), 30-F11 (anti-CD45), 16-10A1 (anti-CD80), GL1 (anti-CD86), M5/114.15.2 (anti-MHC class II), MEL-14 (anti-CD62L), IM7 (anti-CD44), eBioKAT-1 (anti-intercellular adhesion molecule 1 (ICAM-1)), eBioL31 (anti-CD207), RB6-8C5 (anti-Gr-1), 4B12 (anti-CCR7)(eBioscience, San Diego, CA), and G8.8 (anti-EpCAM) (BioLegend, San Diego, CA) were purchased.

For Langerin (CD207) staining, cells were fixed and permeabilized with cytofix/cytoperm solution (BD Biosciences, San Jose, CA), and stained with biotin-conjugated anti-Langerin Ab. Cells were analyzed with FACSCantoII (BD, Franklin Lakes, NJ).

### Histology and immunohistochemistry

Hematoxylin-eosin and toluidine blue staining [31], and the histological scoring were evaluated as reported [19]. In brief, samples were scored for the severity and character of the inflammatory response using a subjective grading scale. Responses were graded as follows: 0, no response; 1, minimal response; 2, mild response; 3, moderate response; and 4, marked response. The slides were blinded, randomized, and reread to determine the histology score. All studies were read by the same pathologist using the same subjective grading scale. The total histology score was calculated as the sum of scores, including inflammation, neutrophils, mononuclear cells, edema, and epithelial hyperplasia.

For immunohistochemistry, cryosections were fixed in acetone, incubated with hamster anti–mouse CD11c (eBioscience) followed by goat anti–hamster AlexaFluor488 (Invitrogen, Carlsbad, CA), and were subsequently incubated with PE-conjugated anti–mouse FcεRIα (eBioscience). Fluorescence images with DAPI staining were obtained using a BIOREVO BZ-9000 system (Keyence, Osaka, Japan).

### Quantitative PCR analysis

Total RNAs were isolated with Trizol (Invitrogen) from ear skin. cDNA was reverse transcribed using a PrimeScript RT reagent kit (Takara Bio, Otsu, Japan). Quantitative RT-PCR with a Light Cycler real time PCR apparatus was performed (Roche Diagnostics, Foster City, CA) using SYBR Green I (Takara Bio). Primers for *Ifng*, *Il17,* and *Il1b* were obtained from Hokkaido System Science (Sapporo, Japan) and the primer sequences were *Ifng,* 5′- ATG AAC GCT ACA CAC TGC ATC -3′ (Forward) and 5′- CCA TCC TTT TGC CAG TTC CTC -3′ (reverse); *Il17*, 5′- TTT AAC TCC CTT GGC GCA AAA -3′ (forward), 5′- CTT TCC CTC CGC ATT GAC AC -3′ (reverse); and *Il1b*, 5′- GCA ACT GTT CCT GAA CTC AAC T -3′ (forward), 5′- ATC TTT TGG GGT CCG TCA ACT -3′ (reverse). For each sample, triplicate test reactions and a control reaction lacking reverse transcriptase were analyzed for expression of the genes and results were normalized to those of the ‘housekeeping’ glyceraldehyde-3-phosphate dehydrogenase (*Gapdh*) levels.

### Lymphocyte proliferation assay and cytokine production

For DNBS-dependent proliferation, single-cell suspensions from skin-draining LNs of mice 5 days after sensitization with DNFB. One million LN cells were cultured with or without 100 µg/ml of DNBS for 72 h, pulsed with 0.5 µCi 3H-thymidine for the last 24 h, and subjected to liquid scintillation counting.

For measurement of cytokine production, the culture supernatants were collected 72 h after incubation and were measured by ELISA (BD Biosciences and R&D systems, Minneapolis, MN) according to the manufacture's protocol.

### CHS protocol

Mice were sensitized with 50 µl of 0.5% (w/v) DNFB in acetone/olive oil (4/1) or 2% oxazolone (Wako Pure Chemical Industries, Ltd, Osaka, Japan) in ethanol on abdominal skin. On day 5, the ears were challenged by application of 20 µl of 0.3% DNFB or 1% oxazolone.

For adoptive transfer, LN cells were prepared from the inguinal and axillary LNs of one mouse sensitized with DNFB 5 days previously, and transferred intravenously into a mouse. The ears of these animals were challenged with 20 µl of 0.3% DNFB 1 h later, and the ear thickness change was measured. For adoptive transfer of T cells, T cells purified with CD90.2^+^ microbeads (Miltenyi Biotec, Bergisch Gladbach, Germany) were prepared from the inguinal and axillary LNs of a mouse sensitized with 2% oxazolone 5 days previously, and transferred intravenously into a mouse. The ears of these animals were challenged with 20 µl of 1% oxazolone 1 h later, and the ear thickness change was measured.

### Generation of BMDC and BMMC

Complete RPMI (cRPMI), RPMI 1640 medium (Sigma, St. Louis, MO) containing 10% fetal calf serum (Invitrogen), was used as culture medium. For BMDC induction, 5×10^6^ BM cells were cultured supplemented with 10 ng/ml recombinant murine GM-CSF (PeproTech, Rocky Hill, NJ) for five days [20] (>90% expressed CD11c).

For BMMC induction, 1×10^6^ BM cells were cultured supplemented with 5 ng/ml recombinant murine SCF and IL-3 (PeproTech) for more than three weeks (>98% expressed c-kit and FcεRIα).

For organ culture assay, the skin from mouse ears was split along with cartilage, and the dorsal ear skin without cartilage was floated in a dermal side-down manner in 24-well tissue culture plates. Twenty-four hours later, the cells in the wells were collected for analysis.

### Chemotaxis assay and FITC-induced cutaneous DC migration

Cells were tested for transmigration to CCL21 (R&D Systems) or medium in the lower chamber across uncoated 5-µm transwell filters (Corning Costar Corp., Corning, NY) for 6 h and were enumerated by flow cytometry.

For FITC-induced cutaneous DC migration, mice were painted on the shaved abdomen with 100 µl of 2% FITC (Sigma) dissolved in a 1∶1 (v/v) acetone/dibutyl phthalate (Sigma) mixture, and the number of migrated cutaneous DCs into draining LNs was enumerated by flow cytometry.

### Co-culture of BMDCs and BMMCs

BMDCs and starved BMMCs were co-cultured at a density of 2×10^5^ DCs in 200 µl per well in a 96-well microplate at a DC:MC ratio of 2∶1 and the co-culture was performed for 24 h. Separation of BMDCs and BMMCs was performed by using transwell culture plates with a 3-µm pore size (Costar, Corning). To observe cell-cell contact *in vitro*, BMDCs and BMMCs were co-cultured on poly-L-lysine coated glass coverslips (ASAHI GLASS Co., LTD, Tokyo, Japan) for 24 h and stained with FITC-conjugated anti-c-Kit and PE-conjugated anti-MHC class II.

For inhibition assays, BMDCs and starved BMMCs were co-cultured with or without 5 µg/ml of isotype control Ab (Rat IgG2b, eBioscience), 20 µg/ml of anti-ICAM-1 Ab (YN1/1.7.4, eBioscience) or 5 µg/ml of anti-TNF-α Ab (MP6-XT22, eBioscience) for 24 h. Starved BMMCs were pretreated with or without wortmannin (100 nM; Sigma) or H89 (10 mM; Sigma) for 1 h and then co-cultured with BMDCs for 24 h.

For detection of membrane-bound TNF-α, starved BMMCs were cultured for 24 h with or without non-stimulated or stimulated BMDCs with 100 ng/ml of LPS (Sigma) and 50 ng/ml of CCL21 (R&D systems) for 1 h.

### Cytoplasmic Ca^2+^ imaging

BMMCs were incubated with 5 mM Quest Fluo-8 AM (ABD Bioquest, CA, USA), and BMDCs were stimulated with 100 ng/ml of LPS and 50 ng/ml of CCL21 for 1 h and stained with 2.5 nM tetramethylrhodamine ethyl ester (TMRE) (Invitrogen). The Fluo-8 image and the transmission image were recorded every 10 sec using a back-thinned electron multiplier CCD camera (ImagEM, Hamamatsu Photonics, Japan) and microscope (Eclipse Ti, Nikon, Japan). The fluorescence intensity was expressed as a ratio to the initial value after subtracting background fluorescence.

### Statistical analysis

Unless otherwise indicated, data are presented as the means ± standard deviation (SD) and are a representative of three independent experiments. *P*-values were calculated with the two-tailed Student's *t*-test or one-way ANOVA followed by the Dunnett multiple comparison test. *P* values less than 0.05 are considered to be significantly different between MasTRECK and corresponding WT mice and are shown as * in the figures.

## Supporting Information

Figure S1 **Effect of DT on MC in Mas-TRECK Tg mice.** (A) Skin MCs in Mas-TRECK Tg mice were stained with toluidine blue with (left panel) or without (right panel) DT treatment. The numbers of skin MCs in Mas-TRECK Tg mice with or without DT treatment under steady state or inflammatory conditions (after 24 hours CHS response) are shown (lower). n.d., not detected (n = 5). (B) WT and Mas-TRECK Tg mice (*n* = 5) were treated with DT, and DCs (CD11c^+^), B cells (B220^+^), NK cells (DX5^+^FcεRI^−^), NKT cells (CD3^+^DX5^+^), CD4^+^ T cells (CD3^+^CD4^+^), CD8^+^ T cells (CD3^+^CD8^+^), eosinophils and neutrophils (Gr-1^+^) were obtained from PBMCs 12 days later (left). WT and Mas-TRECK Tg mice (*n* = 5) were treated with DT, and the numbers of basophils (DX5^+^FcεRI^+^) per ml in PBMCs were evaluated 5 days and 12 days later (right). All data are presented as the mean ± SD and are representative of three experiments.
(PDF)

Figure S2 **CHS responses in** ***Kit ^W/Wv^*** **and Bas-TRECK mice.** (A) DNFB-induced CHS in WBB6F1-*Kit*+*/*+ and WBB6F1-*Kit Kit ^W/Wv^* mice. WBB6F1-*Kit*+*/*+ and WBB6F1-*Kit Kit ^W/Wv^* mice were sensitized with or without DNFB and the ear swelling was measured 24 and 48 h after challenge with DNFB (*n* = 10 per group). (B) DNFB-induced CHS in DT-treated WT and Bas-TRECK Tg mice. DT-treated WT and Bas-TRECK Tg mice were sensitized with or without DNFB and the ear swelling was measured 24 and 48 h after challenge with DNFB (*n* = 10 per group). (C) Oxazolone-induced CHS in DT-treated WT and Mas-TRECK Tg mice. Mice were sensitized with 5% oxazolone and challenged with 1% oxazolone at the high hapten dose (*n* = 10 per group). (D) CHS induced by adoptive transfer of CD90.2^+^ T cells from WT and Mas-TRECK Tg mice sensitized with DNFB (*n* = 6 per group). (E) Toluidine blue positive mast cells in the skin with Mas TRECK Tg mice or B6 wild type mice after one hour injection of BMMC (n = 3). All data are presented as the mean ± SD and are representative of three experiments. ^*^, *P*<0.05 versus a corresponding group.
(PDF)

Figure S3 **Decreased infiltrating cells and cytokines in the skin of Mas-TRECK Tg mice after challenge.** (A) The numbers of CD4^+^ T cells, CD8^+^ T cells and neutrophils (CD45^+^Gr-1^high^) in DNFB-challenged skin were counted in DT-treated Mas-TRECK Tg or WT mice (*n* = 5 per group) using flow cytometry. (B) mRNA levels of IFN-γ, IL-17 and IL-1β in the skin after CHS (*n* = 5 per group). All data are presented as the mean ± SD and are representative of three experiments.
(PDF)

Figure S4 **Impaired development of the Th1 subset in CHS and decreased cytokine production in the LN of sensitized Mas-TRECK Tg mice.** (A) Skin-draining LN cells were collected from MaS TRECK Tg and WT mice 5 days after DNFB application. The numbers of CD44^high^CD62L^+^central memory (CM) or CD44^high^CD62L^−^ effector memory (EM) cells and total CD4^+^ and CD8^+^ T cells in the draining LNs with or without sensitization are shown. (B-D) DNBS-induced lymphocyte proliferation (B) and cytokine production (C, D). Cells were collected from MaS TRECK Tg and WT mice 5 days after DNFB application and cultured for 3 days with or without 100 µg/mL DNBS. Cell proliferation was measured by ^3^H-thymidine incorporation. The amounts of IFN-γ and IL-17 in the culture medium were measured by ELISA. *n* = 10 mice per group.
(PDF)

Figure S5 **Attenuated DC maturation in the absence of MCs.** (A) Representative flow cytometry profiles of the skin from WT and Mas TRECK Tg mice (left), and histogram of CD40 expression on LCs and dDCs from DT-treated WT mice and Mas-TRECK Tg mice treated with or without DT. (B) The expression levels of CD40, CD80, and CD86 on LCs and dDCs in the ear skin from WBB6F1-*Kit*+*/*+, –*Kit ^W/Wv^,*WT, and Mas-TRECK Tg mice under steady state conditions.
(PDF)

Figure S6 **timulation of DCs by MCs is dependent on ICAM-1-LFA-1 interaction and on MC membrane-bound TNF-α.** (A, B) Histogram. The expression levels of CD40 on BMDCs co-cultured with BMMCs with isotype control Ab, anti-ICAM-1 Ab, or anti-TNF-α Ab (TNF) (A), or co-cultured with BMMCs that were pretreated with or without wortmannin (WM) or H89 (B). (C) The expression levels of CD40, CD80, and CD86 on BMDCs co-cultured with WT derived BMMCs or TNF-α KO (TNF KO) derived BMMCs.
(PDF)

Video S1 **Quest Fluo-8 AM-stained BMMCs and TMRE-stained BMMCs were mixed on glass coverslips and recorded every 10 sec at ∼30°C using a back-thinned electron multiplier CCD camera.**
(MOV)

Video S2 **Quest Fluo-8 AM-stained BMMCs and TMRE-stained stimulated BMMCs were mixed on glass coverslips and recorded every 10 sec at ∼30°C using a back-thinned electron multiplier CCD camera.**
(MOV)
