# Supplementary figures and images for: Requirement of Interaction between Mast Cells and Skin Dendritic Cells to Establish Contact Hypersensitivity
Source: PLoS One. 2011 Sep 30;6(9):e25538. doi: 10.1371/journal.pone.0025538 (PMC3184129; doi:10.1371/journal.pone.0025538)

Figure S1

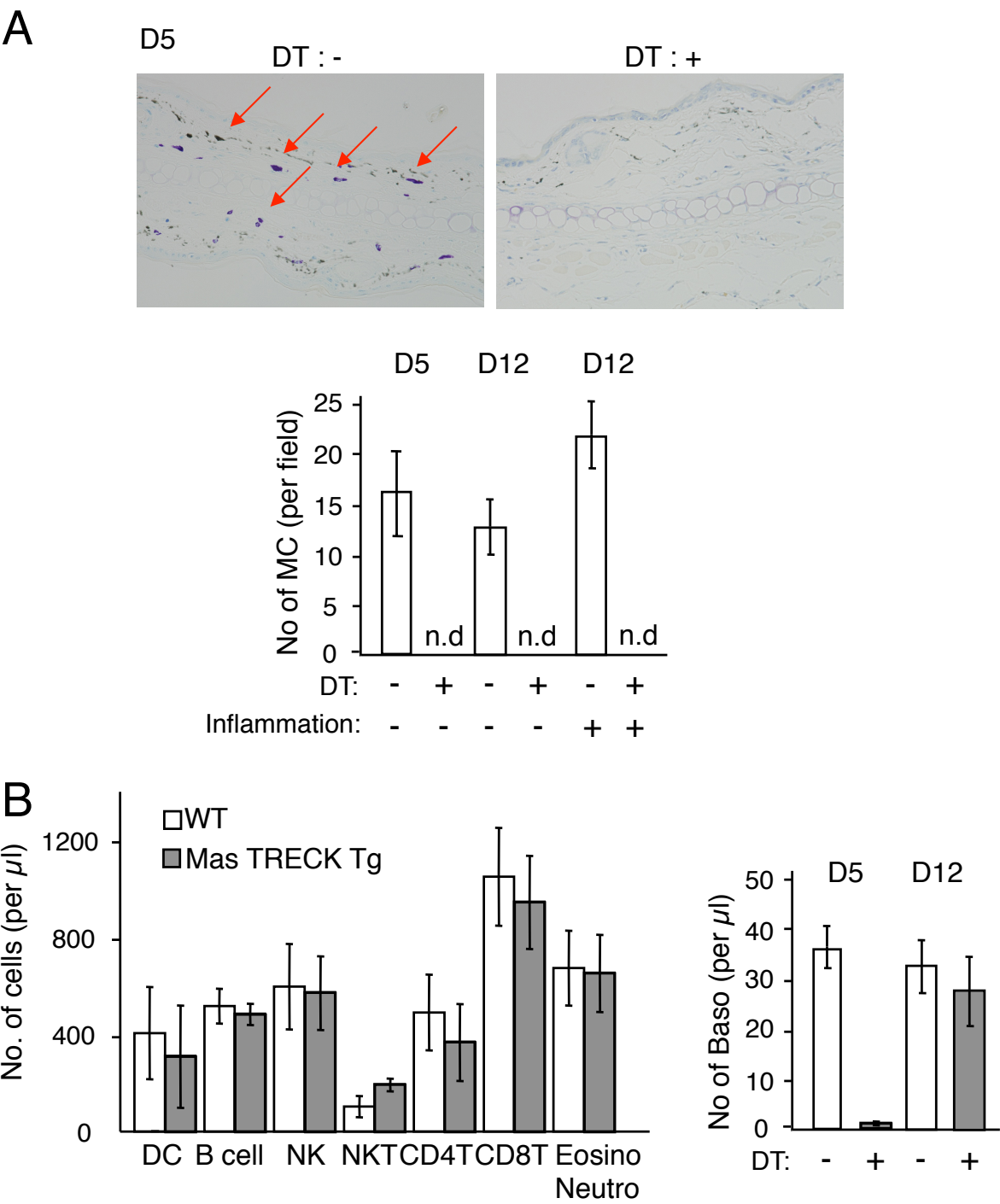

Supplement: Figure S1 — Effect of DT on MC in Mas-TRECK Tg mice. (A) Skin MCs in Mas-TRECK Tg mice were stained with toluidine blue with (left panel) or without (right panel) DT treatment. The numbers of skin MCs in Mas-TRECK Tg mice with or without DT treatment under steady state or inflammatory conditions (after 24 hours CHS response) are shown (lower). n.d., not detected (n = 5). (B) WT and Mas-TRECK Tg mice (n = 5) were treated with DT, and DCs (CD11c+), B cells (B220+), NK cells (DX5+FcεRI−), NKT cells (CD3+DX5+), CD4+ T cells (CD3+CD4+), CD8+ T cells (CD3+CD8+), eosinophils and neutrophils (Gr-1+) were obtained from PBMCs 12 days later (left). WT and Mas-TRECK Tg mice (n = 5) were treated with DT, and the numbers of basophils (DX5+FcεRI+) per ml in PBMCs were evaluated 5 days and 12 days later (right). All data are presented as the mean ± SD and are representative of three experiments. (PDF) [file pone.0025538.s001.pdf]

Figure S2

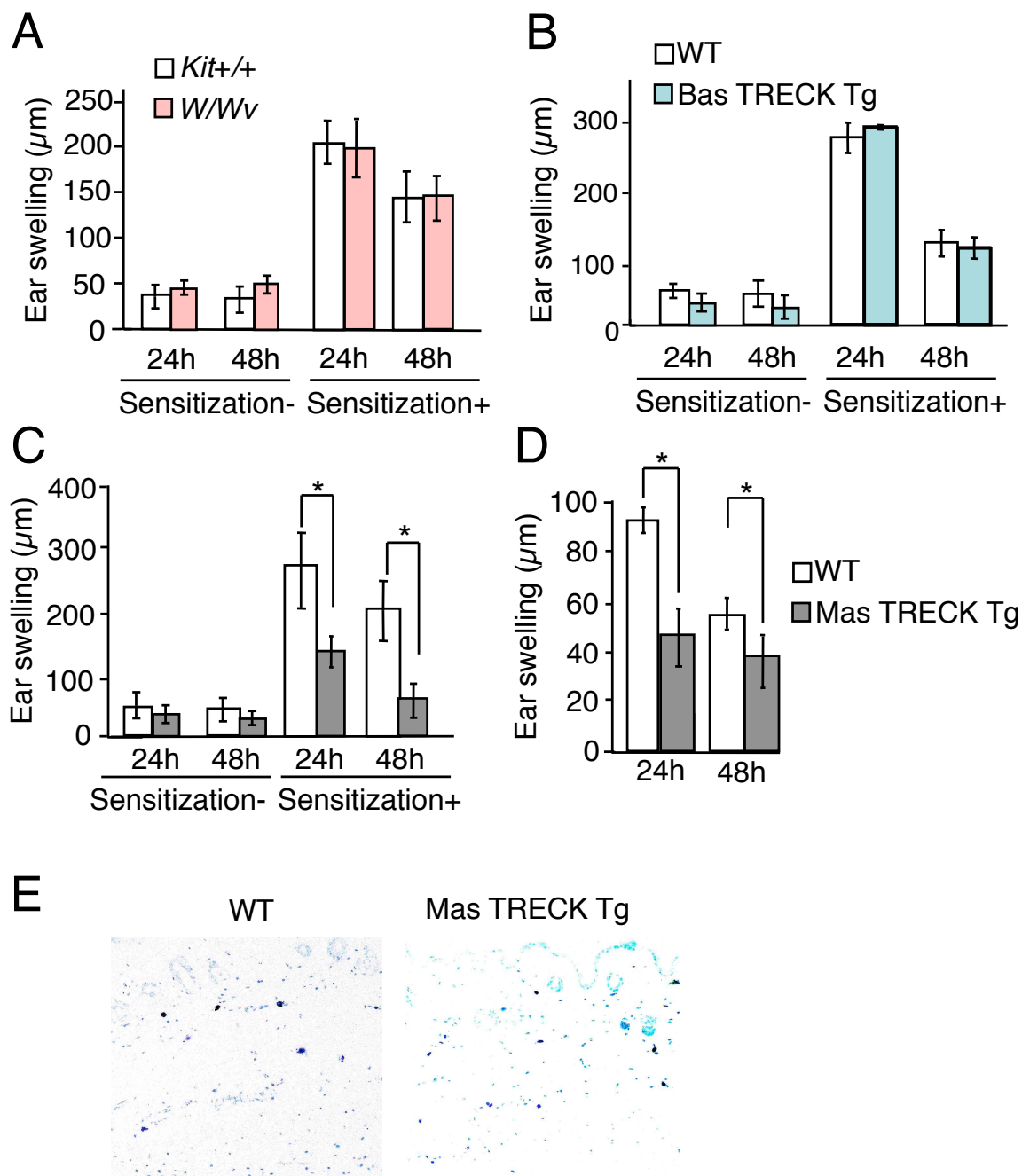

Supplement: Figure S2 — CHS responses in Kit W/Wv and Bas-TRECK mice. (A) DNFB-induced CHS in WBB6F1-Kit+/+ and WBB6F1-Kit Kit W/Wv mice. WBB6F1-Kit+/+ and WBB6F1-Kit Kit W/Wv mice were sensitized with or without DNFB and the ear swelling was measured 24 and 48 h after challenge with DNFB (n = 10 per group). (B) DNFB-induced CHS in DT-treated WT and Bas-TRECK Tg mice. DT-treated WT and Bas-TRECK Tg mice were sensitized with or without DNFB and the ear swelling was measured 24 and 48 h after challenge with DNFB (n = 10 per group). (C) Oxazolone-induced CHS in DT-treated WT and Mas-TRECK Tg mice. Mice were sensitized with 5% oxazolone and challenged with 1% oxazolone at the high hapten dose (n = 10 per group). (D) CHS induced by adoptive transfer of CD90.2+ T cells from WT and Mas-TRECK Tg mice sensitized with DNFB (n = 6 per group). (E) Toluidine blue positive mast cells in the skin with Mas TRECK Tg mice or B6 wild type mice after one hour injection of BMMC (n = 3). All data are presented as the mean ± SD and are representative of three experiments. *, P<0.05 versus a corresponding group. (PDF) [file pone.0025538.s002.pdf]

Figure S3

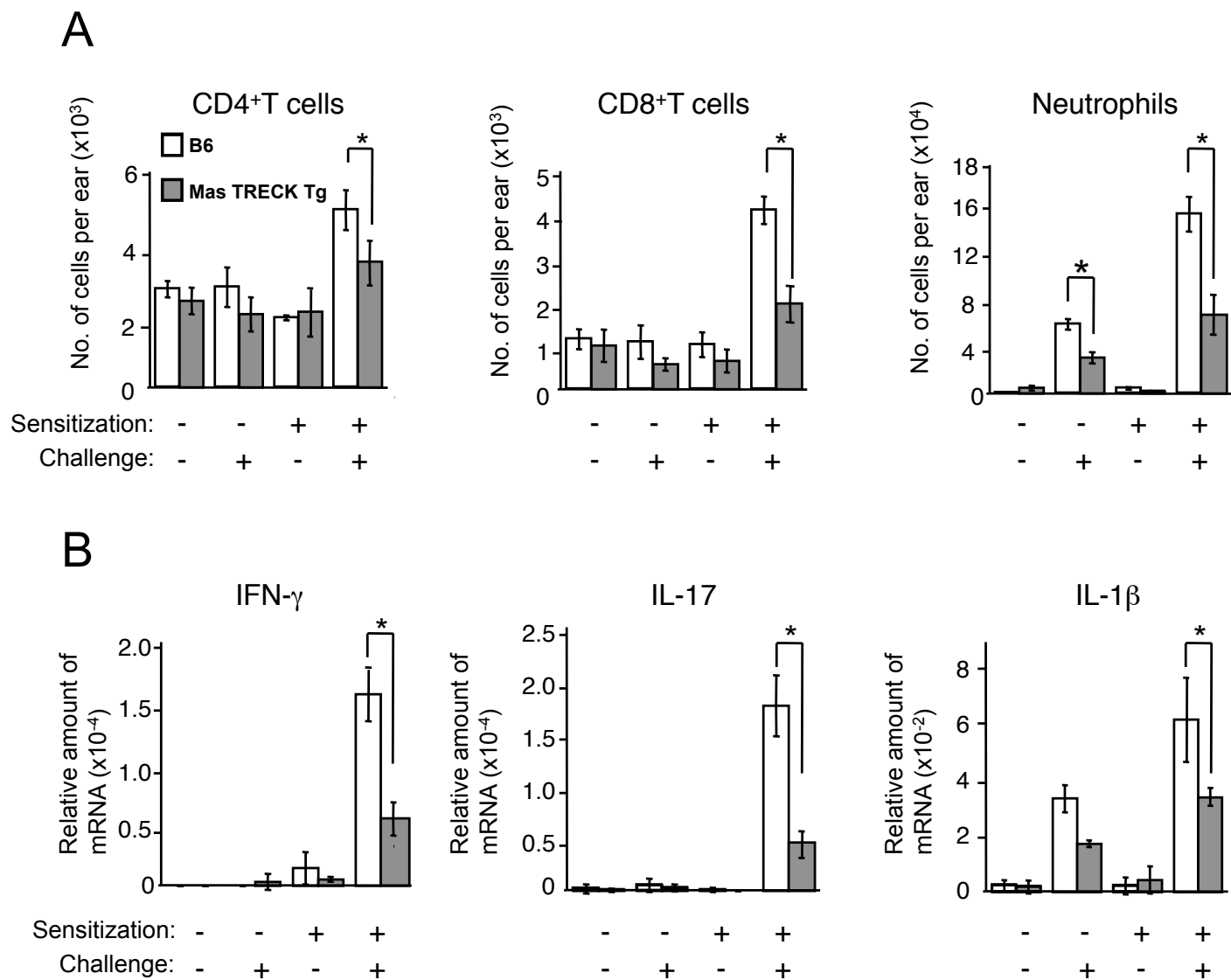

Supplement: Figure S3 — Decreased infiltrating cells and cytokines in the skin of Mas-TRECK Tg mice after challenge. (A) The numbers of CD4+ T cells, CD8+ T cells and neutrophils (CD45+Gr-1high) in DNFB-challenged skin were counted in DT-treated Mas-TRECK Tg or WT mice (n = 5 per group) using flow cytometry. (B) mRNA levels of IFN-γ, IL-17 and IL-1β in the skin after CHS (n = 5 per group). All data are presented as the mean ± SD and are representative of three experiments. (PDF) [file pone.0025538.s003.pdf]

Figure S4

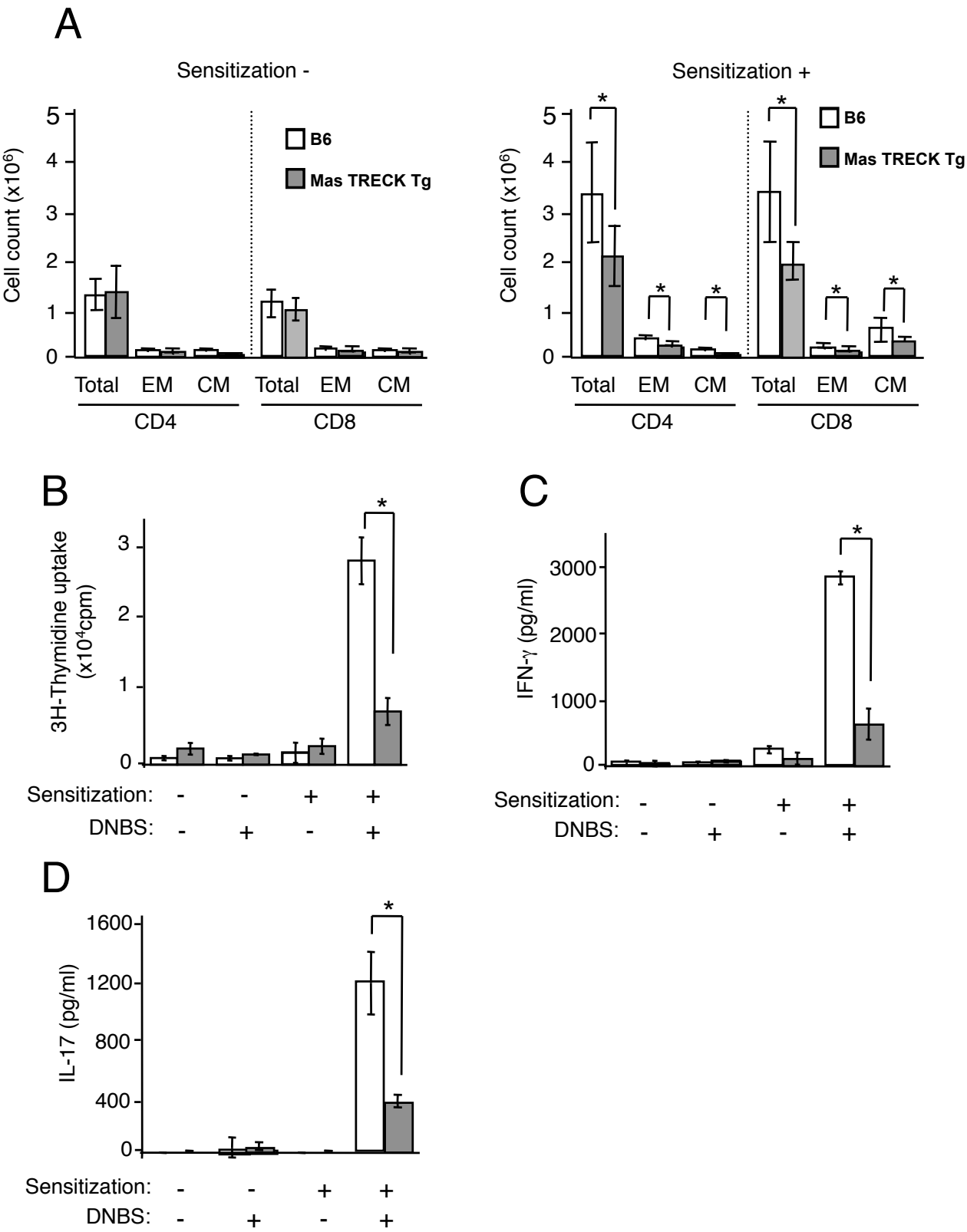

Supplement: Figure S4 — Impaired development of the Th1 subset in CHS and decreased cytokine production in the LN of sensitized Mas-TRECK Tg mice. (A) Skin-draining LN cells were collected from MaS TRECK Tg and WT mice 5 days after DNFB application. The numbers of CD44highCD62L+central memory (CM) or CD44highCD62L− effector memory (EM) cells and total CD4+ and CD8+ T cells in the draining LNs with or without sensitization are shown. (B-D) DNBS-induced lymphocyte proliferation (B) and cytokine production (C, D). Cells were collected from MaS TRECK Tg and WT mice 5 days after DNFB application and cultured for 3 days with or without 100 µg/mL DNBS. Cell proliferation was measured by 3H-thymidine incorporation. The amounts of IFN-γ and IL-17 in the culture medium were measured by ELISA. n = 10 mice per group. (PDF) [file pone.0025538.s004.pdf]

Figure S5

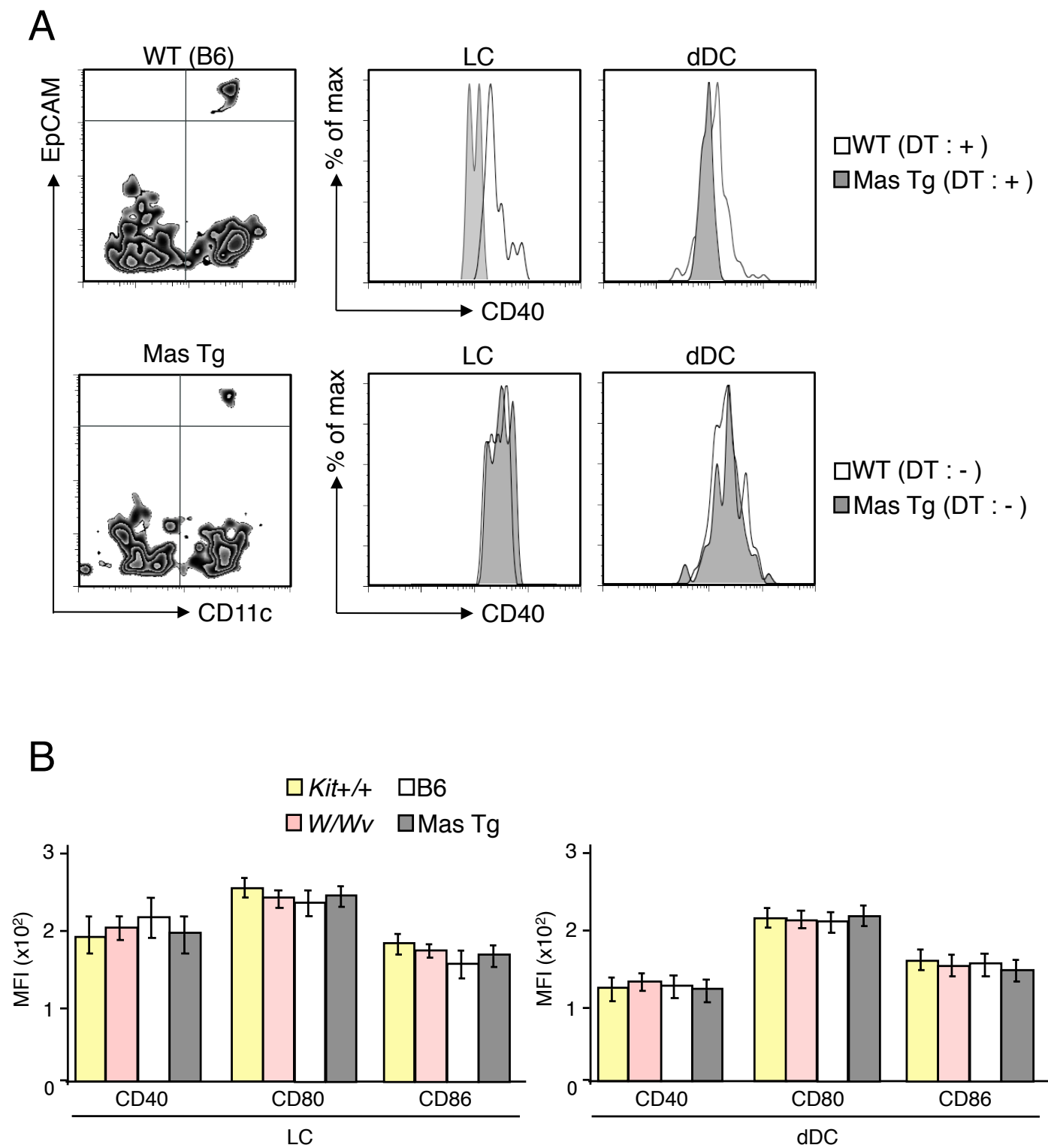

Supplement: Figure S5 — Attenuated DC maturation in the absence of MCs. (A) Representative flow cytometry profiles of the skin from WT and Mas TRECK Tg mice (left), and histogram of CD40 expression on LCs and dDCs from DT-treated WT mice and Mas-TRECK Tg mice treated with or without DT. (B) The expression levels of CD40, CD80, and CD86 on LCs and dDCs in the ear skin from WBB6F1-Kit+/+, –Kit W/Wv,WT, and Mas-TRECK Tg mice under steady state conditions. (PDF) [file pone.0025538.s005.pdf]

Figure S6

A

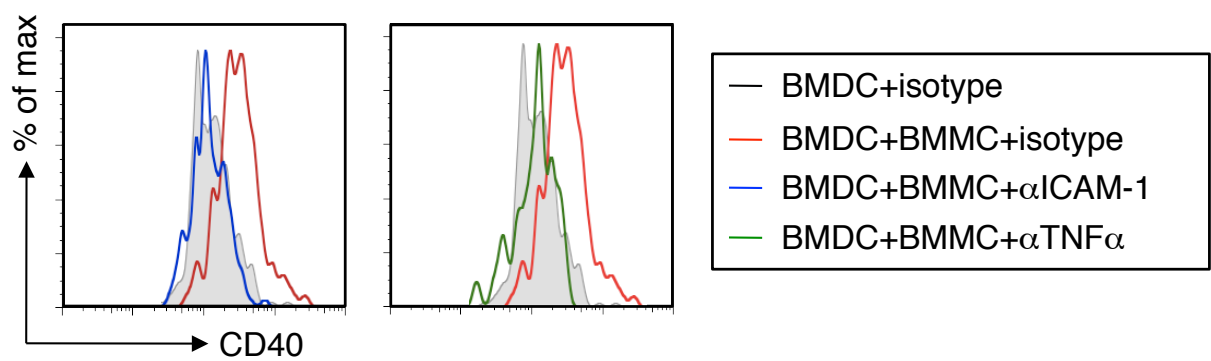

B

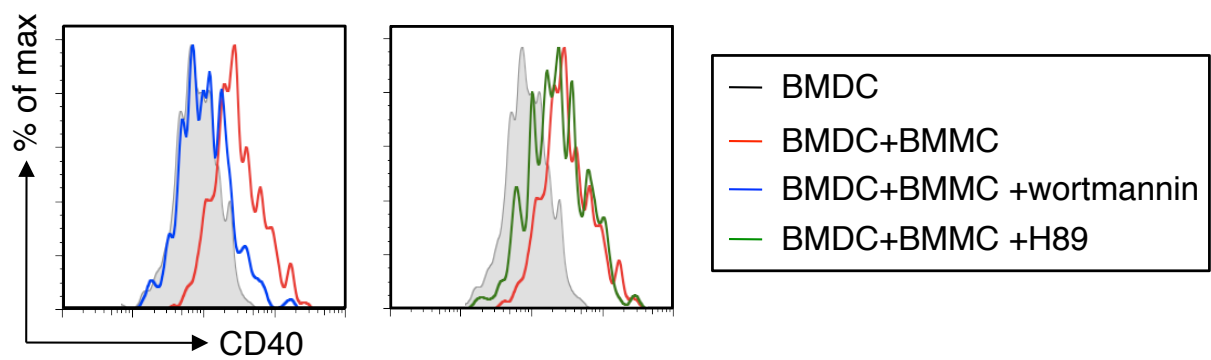

C

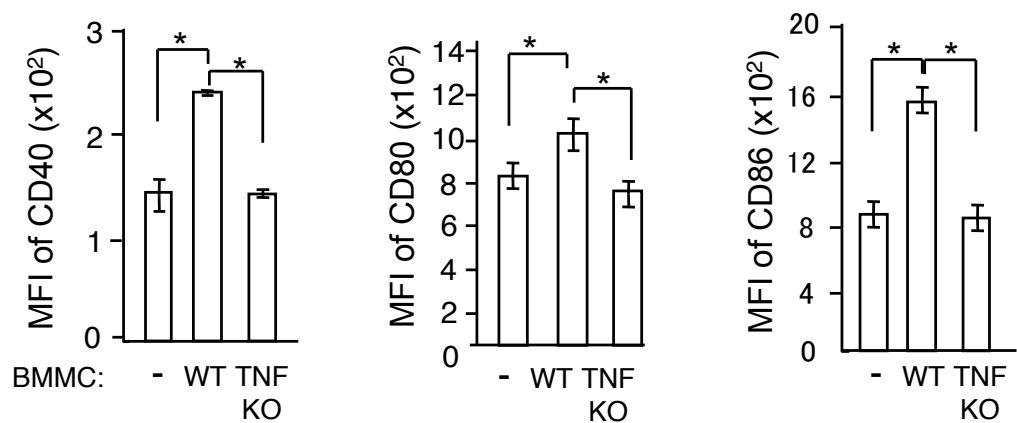

Supplement: Figure S6 — timulation of DCs by MCs is dependent on ICAM-1-LFA-1 interaction and on MC membrane-bound TNF-α. (A, B) Histogram. The expression levels of CD40 on BMDCs co-cultured with BMMCs with isotype control Ab, anti-ICAM-1 Ab, or anti-TNF-α Ab (TNF) (A), or co-cultured with BMMCs that were pretreated with or without wortmannin (WM) or H89 (B). (C) The expression levels of CD40, CD80, and CD86 on BMDCs co-cultured with WT derived BMMCs or TNF-α KO (TNF KO) derived BMMCs. (PDF) [file pone.0025538.s006.pdf]
